# Community-based surveillance of *Cryptosporidium* in the indigenous community of Boliwong, Philippines: from April to December 2017

**DOI:** 10.4178/epih.e2018047

**Published:** 2018-09-28

**Authors:** Ryan V. Labana, Julieta Z. Dungca, Veeranoot Nissapatorn

**Affiliations:** 1 Department of Biology, Polytechnic University of the Philippines, Manila, Philippines College of Science, Philippines; 2 Graduate School, Centro Escolar University, Manila, Philippines; 3 School of Science and Technology, Centro Escolar University, Manila, Philippines; 4 School of Allied Health Sciences (Southeast Asia Water Team), Walailak University, Nakhon Si Thammarat, Thailand; 5 Research Excellence Center for Innovation and Health Products (RECIHP), Walailak University, Nakhon Si Thammarat, Thailand

**Keywords:** *Cryptosporidium*, Epidemiology, Indigenous peoples, Protozoan, Public health, Zoonosis

## Abstract

**OBJECTIVES:**

For the first time, Boliwong, an indigenous community in the Philippines, was surveyed for the prevalence of *Cryptosporidium* from April to December 2017.

**METHODS:**

*Cryptosporidium* oocysts were detected in samples from the river, creek, and water pumps via immunomagnetic separation techniques, and from human and animal concentrated faecal samples using the modified Ziehl-Neelsen technique.

**RESULTS:**

Seven of the 24 water samples (29.2%) were positive for *Cryptosporidium*, with the highest concentration (0.8 oocyst/L) detected in the creek. Of 35 fecal samples from different animal groups, 8 (21.6%) were positive for *Cryptosporidium* oocysts. The highest intensity of oocyst shedding was detected in dogs (χ^2^ =8.00). Of the 137 human fecal samples, 39 (28.5%) were infected with *Cryptosporidium*. In this study, 3 risk factors were found to be associated with infection: (1) location (crude odds ratio [cOR], 16.39; 95% confidence interval [CI], 2.11 to 127.41; p=0.008), (2) drinking water from the natural spring (cOR, 0.29; 95% CI, 0.11 to 0.82; p<0.05), and (3) using an open pit as a sanitary toilet facility (cOR, 2.44; 95% CI, 1.14 to 5.20; p<0.05). When the cOR was adjusted, using an open pit as a sanitary toilet facility remained a significant risk factor of infection (adjusted OR, 0.41; 95% CI, 0.19 to 0.90; p<0.05).

**CONCLUSIONS:**

There is a potentially emerging *Cryptosporidium* zoonosis in Boliwong, Lagawe, Philippines. It is recommended that the toilet facilities and the water system in the community be rehabilitated to avoid any possible disease outbreak. Health education is also needed in the community to maintain proper hygiene and sanitation practices.

## INTRODUCTION

Two million people across the globe die every year due to waterborne diseases [1]. *Cryptosporidium*, a waterborne parasite in the subphylum Apicomplexa, is recognized as a major cause of the increasing number of water-associated outbreaks in the past 10 years [2-4]. It is transmitted through the intake of food and water contaminated with transmissible oocysts [5]. Transmission also occurs through direct contact with an infected human or animal and contaminated water, faeces, soil, or vegetation. *Cryptosporidium* infects a wide range of human and animal hosts, and the density of their infective oocysts is sufficient to pollute the aquatic environment, promoting a wide-range of zoonotic infections [6]. Since it is ubiquitous, and because the infective oocysts are microscopic (as small as 2 μm) and can pass through traditional filtration systems, *Cryptosporidium* is now considered as an important public health concern. It causes symptoms of watery diarrhoea, abdominal pain, weight loss, vomiting, fatigue, fever, and even morbidity and death [7].

In the Philippines, 55 people die every day from underdiagnosed diseases due to contaminated water [8]. As an emerging market economy, the Philippines are undergoing continuous industrialization and urbanization [9]. These factors are commonly associated with the issues involving accessibility of clean water that are currently confronting the country. The effluent from developments that are part of modernization typically impacts the nearby rural communities. In addition, agricultural runoff and domestic discharge in rural areas carry raw sewage, fertilizers, heavy metals, and solid wastes to the receiving bodies of water [10]. These environmental changes, together with the population explosion in rural communities and the effects of closer contacts between people and domestic animals, have altered the ecological balance between pathogens and their animal and human hosts [11].

*Cryptosporidium* is not well studied in the Philippines. Since 1985, when it was first reported in diarrheic patients in the country [12], few studies have been conducted of its prevalence and occurrence. No study has investigated its epidemiology. Recently, there have been reports of a high prevalence of *Cryptosporidium* in treated and untreated water sources in various regions of Luzon [13]. An alarming prevalence of 77.3% was also reported in a study 2conducted in the Metropolitan Waterworks and Sewage System, a major water source for the majority of Filipinos in Metro Manila and neighbouring provinces [14]. In a different study, oocysts were detected in all (100%) the water samples collected from public swimming pools in Laguna [15]. Despite these alarming findings, *Cryptosporidium* remains underappreciated as a threat to public health in the Philippines.

This study was conducted to understand the possible role of *Cryptosporidium* in the emergence of waterborne diseases in the Philippines. It aimed to draw conclusions from a single community in the Cordillera Administrative Region (CAR) with the goal of using the findings as a potential epidemiologic model for other indigenous communities in the country. CAR is a region in the Philippines with a persistently high prevalence of diarrhoea. Several municipalities in this region have been reported to have poor water and sanitation conditions; the toilets are mostly open pits, while the water sources are mostly deep well and/or spring developments [16,17]. This study further aimed to identify risk factors of *Cryptosporidium* infection and to estimate the points of oocyst transmission to help policy-makers formulate sensitive and effective community health programs.

## MATERIALS AND METHODS

### Study location and population

**Figure 1.**
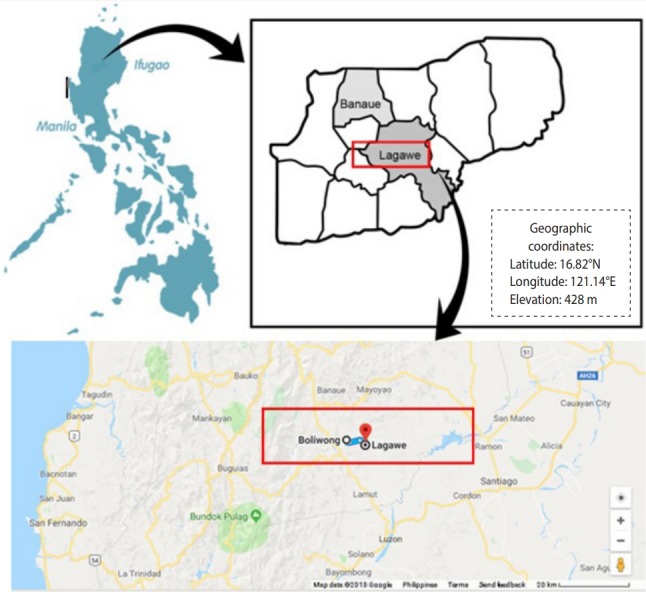
Map of Boliwong in Lagawe, Philippines.

The cross-sectional study was conducted from April to December 2017 in an indigenous community inhabited by the Tuwalis, an ethnolinguistic group in Boliwong, Lagawe, Philippines (16.8221°N, 121.1424°E). This area is covered by high mountains with a total land area of 956.4726 ha and an elevation of 563.2 m above mean sea level. The community was reported to have poor sanitation, unimproved water sources, and low socio-demographic status. Figure 1 shows the location of Boliwong in the northeast part of the Philippines.

Stratified random sampling was performed by forming 3 strata in the study location: (1) lowland, (2) midland, and (3) upland. From the lowland region, 30 consenting participants were drawn from 560 population members (5.3%), and the corresponding participation rates from the midland and upland regions were 35 of 550 (6.4%) and 72 of 570 (12.6%), respectively. The total sample was 137 (8.1%) of the total population of 1,680 in Boliwong. Participants were randomly chosen with substitution for data gathering and faecal sample collection.

A standardized questionnaire, probing general information, household income, water sources, sanitation, and hygiene practices, was distributed among participating heads of households or adults over 18 years old. The participants were asked to sign the consent form after indicating that they understood the main objective of the study.

### Collection of human and animal faecal samples

To complete the participation, each participant was asked to submit a faecal sample. A spatula that came with the container was used to place the human faecal sample in a clean screw-top container. The participants were advised to fill the container with a pea-sized sample, without placing it into contact with urine and water from the toilet. Faecal samples were also collected from domestic dogs (n=12), domestic cats (n=7), domestic swine (n=4), goats (n=6), carabaos (n=2), and chicken (n=6). If defecation was not directly observed, the presence of high-moisture sheen on the surface of the faeces was observed to ensure faecal freshness. All samples were collected from surfaces that had not been in contact with the substratum to avoid contamination.

### Detection of *Cryptosporidium* via modified Ziehl-Neelsen staining

To detect *Cryptosporidium* oocysts from the animal and human faecal samples, modified Ziehl-Neelsen staining was done. This method has already been established for the detection of *Cryptosporidium* oocysts. Briefly, faecal smears were made from the concentrated deposit. It was air-dried, fixed in methanol for 3 minutes, and stained with strong carbol fuchsin for 15-20 minutes. Tap water was used for thorough rinsing, followed by decolorizing in acid alcohol (1.0% hydrochloric acid [HCl] in methanol) for 15-20 seconds. The sample was rinsed thoroughly again in tap water and was counterstained with 0.4% malachite green for 30-60 seconds. After final rinsing and air drying, the sample was examined with cedarwood oil under the oil immersion objective [18]. A zigzag method was used to view the fields of the cover slip. Pink or red stains corresponded to *Cryptosporidium* oocysts. The oocysts were measured, and ranged from 4 to 6 μm in diameter. Morphological details, including non-homogeneous colour and the appearance of a hollow portion of the oocyst, were also observed. Any particle that did not meet these criteria was excluded from the *Cryptosporidium*-positive samples [19].

### Collection, filtration, concentration, and immunofluorescence assay of water samples

A total of 24 surface-grab water samples was collected from the river (n=9), creek (n=9), and water pumps (n=6). The water samples were filtered using a nitrocellulose membrane (47 mm in diameter, 0.2 μm pore size) by mild suction using a vacuum pump. The obtained sediment was carefully washed using 0.1% Tween-80. The washed sediment was centrifuged at 3,000×g for 15 minutes and was aspirated into a 10-mL syringe. To isolate the oocysts, an anti-*Cryptosporidium* bead conjugate was added to the sample, allowing the oocysts to bind with the beads. The tube was then placed in a magnetic particle concentrator (MPC-1) to magnetize the sample and separate the oocyst-magnetic complex from the contaminating debris. The supernatant was then removed and discarded. The beads were separated again with 50 μL of 0.1 N HCl using a MPC-M. Each sample concentrate was placed in the well of a microscope slide. When dried, it was fixed with methanol and was labelled with anti-*Cryptosporidium* fluorescein isothiocyanate (FITC) monoclonal antibody. The assay was carried out using a commercial kit Dynabeads GC-Combo (Thermo Fisher Scientific, Waltham, MA, USA). *Cryptosporidium* oocysts were recognized based on fluorescence. Round or oval shapes with bright apple-green fluorescence that measured 4 to 6 μm were confirmed as *Cryptosporidium*. To confirm the presence of nuclei in the oocysts, light or intense blue internal staining was observed upon examination using 4′, 6-diamidino-2-phenylindole of the samples identified as positive based on the FITC examination.

### Statistical analysis

The prevalence rate was computed by dividing the number of parasite-positive human/animal faecal samples by the total number of samples collected within each risk-factor category/animal group. SPSS version 23.0 (IBM Corp., Armonk, NY, USA) was used to analyse the data. Univariate logistic regression analysis evaluated the association between each risk factor (e.g., location, age, sex, water source, and sanitation- and hygiene-related factors) and *Cryptosporidium* infection. The results were calculated as crude odds ratios (cORs). Risk factors at p-value <0.05 were considered significant and were subjected to a multivariate regression analysis to further clarify their associations. The intensity of oocyst shedding among the animal groups was determined by computing the mean count of oocysts per animal group and their respective 95% confidence intervals (CIs).

## RESULTS

The results of this study showed that *Cryptosporidium* oocysts were present across the water sources, animals, and human inhabitants in Boliwong. Of the 137 human faecal samples, 39 (28.5%) were infected with *Cryptosporidium* oocysts from participants who reported no symptoms of infection (76.9%). Seven (17.9%) participants reported having diarrhoea in the past six months, 2 (5.1%) had amoebiasis, 1 (2.6%) had gastroenteritis, and 1 other participant (2.6%) reported a case of vomiting.

Of the 37 animal faecal samples, 8 (21.6%) harboured *Cryptosporidium*. The highest prevalence of *Cryptosporidium* was observed in dogs and chickens, both of which had a prevalence of 33.3%, followed by pigs and goats, with a prevalence of 25.0 and 16.7%, respectively. However, no oocysts were detected from cats or carabaos. The results of the prevalence of *Cryptosporidium* from human and animal faecal samples are shown in Table 1.

**Figure 2.**
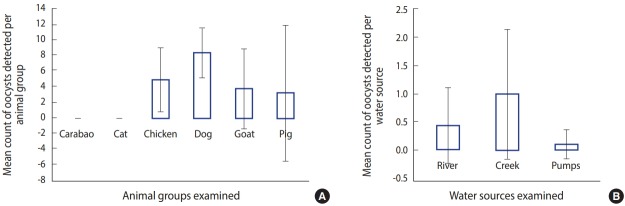
Mean count of oocysts among (A) animal groups and (B) water sources.

Of the positive animal samples, those from dogs had the highest intensity (Figure 2). The conjecture of emerging zoonosis in the community was reinforced when the intensity of oocysts was determined both in animal faecal and water samples. Seven (29.2%) of the 24 water samples were positive for *Cryptosporidium* oocysts (data not shown). The oocysts were detected from all the examined water sources (i.e., the river, creek, and water pumps). The highest concentration was detected in the creek (0.8 oocyst/L), while the lowest concentration was detected in water pumps (0.1 oocyst/L). The concentration of *Cryptosporidium* in the river was 0.4 oocyst/L.

To fully understand the emerging *Cryptosporidium* zoonosis in Boliwong, hygiene and sanitation practices were also investigated (Table 2). Three risk factors were deemed to be significantly associated with *Cryptosporidium* infections in the community: (1) location, (2) drinking water from the natural spring, and (3) using an open pit for defecation. The majority of the participants reported getting water from the natural spring for drinking consumption (72.3%) without any treatment, whilst the rest (27.7%) purchased bottled water from water refilling stations in Poblacion East, the capital of Lagawe. Water from the natural spring was distributed to the respective households through a piping system. There was no recorded regular monitoring of pipes in the community, so the quality of drinking water was not ensured. Drinking water from the natural spring was identified as a significant risk factor in this study (cOR, 0.29; 95% CI, 0.11 to 0.82; p<0.05).

In regard to sanitation, 56 of the 137 participants (40.9%) had no sanitary toilet facilities, while 81 (59.1%) had a flush or pour latrine in their household. The prevalence of *Cryptosporidium* infection was higher among those who had no improved toilet facility (39.3%) than among their counterparts (21.0%). Using an open pit for defecation was also significantly associated with *Cryptosporidium* infection (cOR, 2.44; 95% CI, 1.14 to 5.20; p<0.05).

Another significant risk factor determined in this study was the location. The results showed that people living in the upland and midland regions of Boliwong had higher odds of *Cryptosporidium* infection. The standard of living of the people in the upland and midland regions was determined to be low. Most of the houses in the upland and midland regions were made of light materials and were always open for pet animals such as cats and dogs. The children habitually played on the ground with no footwear. There was also a garbage dump site situated in the upland region that was accessible to children playing in its vicinity. These conditions placed individuals at risk of parasitic infections. In contrast, the lowland area was less rural and less populated than the upland and midland areas. When the cORs of the significant risk factor were adjusted, using an open pit for defecation remained associated with *Cryptosporidium* infection.

**Figure 3.**
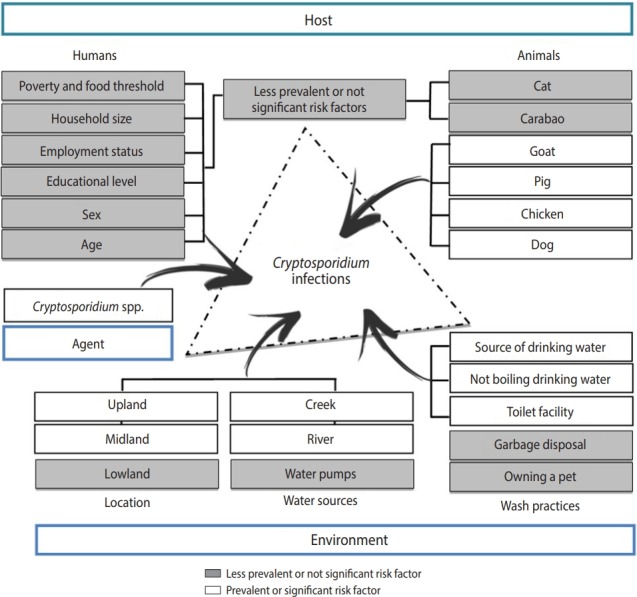
Epidemiologic triad of *Cryptosporidium* infection in Boliwong.

The interconnections of the human, animal, and environmental interface in the community of Boliwong were characterized in this study through a simple epidemiologic triad (Figure 3). The epidemiologic triad was designed to promote the One Health approach in treating *Cryptosporidium* occurrence in the community, as this approach is an increasingly important prismatic view of the multifactorial causes of infectious disease. Its main goal is to provide governments, non-governmental organizations, and practitioners with a view of the health problem (in this case, *Cryptosporidium* infection).

## DISCUSSION

This is the first report of *Cryptosporidium* infection in an indigenous community in the Philippines. The findings in this study provide conservative evidence of the potential emergence of *Cryptosporidium* transmission in the community of Boliwong. Our findings revealed the co-existence of human and animal infections in the community capable of triggering further proliferation of oocysts [7].

### Role of the water system in the prevalence of infection

The transmission of *Cryptosporidium* oocysts is usually waterborne. Contaminated water can be a direct source of human infection through drinking or accidental ingestion of contaminated water. Contaminated water can also infect humans if it is used in washing and preparing food, which is considered foodborne transmission [2].

There is one long stretch of river and another stretch of creek in this community. These water systems traverse the upstream, midstream, and downstream regions of Boliwong, serving a large portion of the community. These bodies of water, together with water pumps, are used by the human inhabitants for bathing, swimming, and laundry activities. Aside from domestic use, these also serve as the main water sources of the terraced ponds and rice paddies of the farmers. The creek, for example, is connected to the tubing system in the rice field that is used for watering purposes. A high prevalence of *Cryptosporidium* in the creek was identified in this study, which poses the risk that the oocysts may spread widely, not only to the food of the people in the community, but also to the regions that receive the harvested rice.

Furthermore, various animals have access to the water system—specifically to the river and creek—to drink, bathe, and defecate. Voided goat, carabao, and dog faecal samples were observed less than a meter away from the river. This close proximity between animals and the water system might cause the massive dispersal of oocysts in the environment.

### Animals as an agent of oocyst dispersal

Several animal groups were visibly present in the community. Carabaos and goats were usually tied in the backyard, while pigs were put inside a cage. Of note, most owners of these pets did not practice any manure management. Manure and other pet waste, and even human faeces, visibly pooled on the surrounding ground. The pooling of animal faeces on the ground, with subsequent drainage into waterways, was considered as a significant contributor to infections. Dogs and chickens had the highest intensity of oocyst infection. These animal groups, together with cats, are free roamers in the community, with access to houses, gardens, rice fields, and other places. Birds and dogs are among the non-human hosts of *Cryptosporidium* that are considered reservoirs for human infections because they harbour *C. meleagridis* and *C. canis*, which have been reported to infect humans as well [20]. Several studies have demonstrated contrasts in clinical manifestations among the different species of *Cryptosporidium*. *C. hominis*, for instance, is associated with diarrhea, nausea, and vomiting. C. parvum, *C. meleagridis*, and *C. canis*, in contrast, are only associated with diarrhoea. Furthermore, there are other species that can infect animal hosts, but not necessarily humans [21]. This study did not identify the species of the detected *Cryptosporidium* via molecular techniques. Despite this gap, the potential for *Cryptosporidium* infections in the community can still be considered high due to the important ecological characteristics of the community that favour the widespread transmission of oocysts.

### The threat of *Cryptosporidium* infections in humans

The 28.5% prevalence of *Cryptosporidium* infection among human inhabitants in Boliwong should not be dismissed. Poor hygiene and sanitation, particularly regarding the widespread use of open pits as toilet facilities in the community, can worsen the present condition. People regularly drink water from the natural spring, which possibly harbours the parasite. They are also in direct contact with pets and farm animals that increase the risk of contracting *Cryptosporidium* infection. The habitual use of unsafe water sources (i.e., river, creek, and water pumps) in the community supports the conjecture that *Cryptosporidium* may be emerging as a zoonosis in the community.

Interventions should be made to address the factors found to be influential in the spread of parasitic infections. It is advisable that people in Boliwong should avoid accidental intake of water from the river during bathing and when engaging in other recreational activities. It is also not advisable to have contact with the creek, as it was contaminated with *Cryptosporidium*. The dispersal of animal groups throughout the environment should be minimized by placing chicken in cages and maintaining other pets at a distance. If a person cannot avoid contact with animals, proper handwashing should be a habit.

Furthermore, providing a developed water source and improved toilet facilities to the households in the community is strongly recommended and would make a major positive impact on the prevention of *Cryptosporidium* infections. The people in Boliwong should also understand the various means of transmission of parasites from animals to the environment and to humans. Proper hygiene and sanitation behaviour should be further encouraged in the community through community-wide health education. An One Health approach should be applied to the eradication of parasites, since several sources were identified as infected or contaminated.

An estimated 14-17 million indigenous people inhabit the Philippines [22]. The findings of this study should receive attention and lead to the implementation of similar epidemiological surveillance programs among other indigenous communities in the country. The impact of waterborne pathogens such as *Cryptosporidium* from the public health perspective has not been highlighted in the Philippines due to the paucity of domestic studies regarding this important protozoan parasite. Sufficient data on *Cryptosporidium* in various areas and from different sources could be used by experts to formulate possible solutions.

In conclusion, the people of Boliwong are at risk of contracting *Cryptosporidium* infections, as *Cryptosporidium* may be emerging as a zoonosis in the community. Unimproved water sources and poor sanitation practices support the wide dispersal of oocysts throughout large parts of the community. *Cryptosporidium* oocysts were detected from several key point sources (i.e., humans, animals, and the water system), which calls for consistent interventions before any waterborne outbreak involving *Cryptosporidium* or other relevant intestinal protozoan parasites occurs in the near future.

## Figures and Tables

**Table 1.** Prevalence of *Cryptosporidium* infection in human and animal samples

Fecal sample	n	Infected, n (%)
Human	137	39 (28.5)
Cat	7	0 (0.0)
Dog	12	4 (33.3)
Goat	6	1 (25.0)
Pig	4	1 (25.0)
Carabao	2	0 (0.0)
Chicken	6	2 (33.3)

**Table 2.** Univariate and multivariate analysis of factors associated with *Cryptosporidium* infection

Variables	n	Infected (%)	cOR (95% Cl)	p-value	aOR (95% Cl)	p-value
Location						
Upland	72	36.1	16.39 (2.11, 127.41)	0.01	1.58 (0.65, 3.82)	0.31
Midland	35	34.3	15.13 (1.83, 125.06)	0.01	-	-
Lowland	30	3.3	1.00 (reference)			
Age (yr)						
≤ 12	63	27.0	0.87 (0.41, 1.84)	0.72	-	
>12	74	29.7	1.00 (reference)			
Sex						
Male	63	28.6	1.01 (0.48, 2.13)	0.98	-	
Female	74	28.4	1.00 (reference)			
Size of household (n)						
>5	52	32.7	1.39 (0.65, 2.96)	0.39	-	
≤5	85	25.9	1.00 (reference)			
Poverty threshold						
Below	98	28.6	1.02 (0.45, 2.32)	0.97	-	
Within	39	28.2	1.00 (reference)			
Food threshold						
Below	73	30.1	1.19 (0.57, 2.52)	0.64	-	
Within	64	26.6	1.00 (reference)			
Educational level (attended-finished)						
Elementary	54	36.0	1.14 (0.40, 3.28)	0.80	-	
High school	30	21.4	0.57 (0.16, 2.02)	0.38	-	
College	23	30.4	1.00 (reference)			
Employment status						
Employed	44	35.0	1.69 (0.79, 3.64)	0.18	-	
Unemployed	93	24.0	1.00 (reference)			
Source of drinking water						
Bottled water	37	13.5	0.29 (0.11, 0.82)	0.02	1.48 (0.56, 3.91)	0.42
Natural spring	100	34.0	1.00 (reference)			
Boiling water before consumption						
Yes	84	33.3	1.91 (0.85, 4.27)	0.11	-	
No	53	20.8	1.00 (reference)			
Type of toilet facility						
Open pit	56	39.3	2.44 (1.14, 5.20)	0.02	0.41 (0.19, 0.90)	0.02
Flush/pour	81	21.0	1.00 (reference)			
Garbage disposal						
Garbage truck	20	20.0	MV	-	-	
Burning	100	18.0	MV	-	-	
Open pit	17	100				
Owns a pet						
1-2	97	30.9	1.54 (0.65, 3.64)	0.32	-	
≥3	40	22.5	1.00 (reference)			

cOR, crude odds ratio; CI, confidence interval; aOR, adjusted odds ratio; MV, missing values.
